# A conceptual framework for the emerging discipline of conservation physiology

**DOI:** 10.1093/conphys/cou033

**Published:** 2014-09-24

**Authors:** Laura E. Coristine, Cassandra M. Robillard, Jeremy T. Kerr, Constance M. O'Connor, Dominique Lapointe, Steven J. Cooke

**Affiliations:** 1 Canadian Facility for Ecoinformatics Research, Department of Biology, University of Ottawa, 30 Marie-Curie, Ottawa, ON, Canada K1N 6N5; 2 Department of Psychology, Neuroscience, and Behaviour, McMaster University, 1280 Main Street West, Hamilton, ON, Canada L8S 4L8; 3 Fish Ecology and Conservation Physiology Laboratory, Department of Biology, Carleton University, 1125 Colonel By Drive, Ottawa, ON, Canada K1S 5B6; 4 Institute of Environmental Science, Carleton University, 1125 Colonel By Drive, Ottawa, ON, Canada K1S 5B6

**Keywords:** Ecology, global change, physiological tolerance, policy, resource management, restoration

## Abstract

Current rates of biodiversity decline are unprecedented and largely attributed to anthropogenic influences. Given the scope and magnitude of conservation issues, policy and management interventions must maximize efficiency and efficacy. The relatively new field of conservation physiology reveals the physiological mechanisms associated with population declines, animal–environment relationships and population or species tolerance thresholds, particularly where these relate to anthropogenic factors that necessitate conservation action. We propose a framework that demonstrates an integrative approach between physiology, conservation and policy, where each can inform the design, conduct and implementation of the other. Each junction of the conservation physiology process has the capacity to foster dialogue that contributes to effective implementation, monitoring, assessment and evaluation. This approach enables effective evaluation and implementation of evidence-based conservation policy and management decisions through a process of ongoing refinement, but may require that scientists (from the disciplines of both physiology and conservation) and policy-makers bridge interdisciplinary knowledge gaps. Here, we outline a conceptual framework that can guide and lead developments in conservation physiology, as well as promote innovative research that fosters conservation-motivated policy.

## Abstract (precis)

We present a conceptual framework for conservation physiology intended to facilitate the application of physiological knowledge and concepts to conservation problems. The framework is focused on moving from knowledge to action, (e.g., policy, management interventions), which is essential if this mission-oriented discipline is to contribute meaningfully to evidence-based conservation and management.

## Introduction

Global environmental change is leading to unprecedented levels of biodiversity loss (Rockstrom *et al.*, 2009). Anthropogenic drivers of decline, including habitat alteration (Kerr and Deguise, 2004; Gallant *et al.*, 2007), climate change (Pearson and Dawson, 2005; Monahan and Hijmans, 2008) and pollution (Menezes-Oliveira *et al.*, 2013), perturb the physiological optimization of organisms (Carey, 2005; Pörtner and Farrell, 2008). When unchecked, effects of species decline can cascade through ecosystems and trophic communities (Duffy, 2003), leading to loss of specialist species (White and Kerr, 2007) or even changes in system state (Beisner *et al.*, 2003). The complexity of threats and their concomitant interactions (Brook *et al.*, 2008) require decisive and efficient conservation and management actions (Knight *et al.*, 2006).

**Figure 1:**
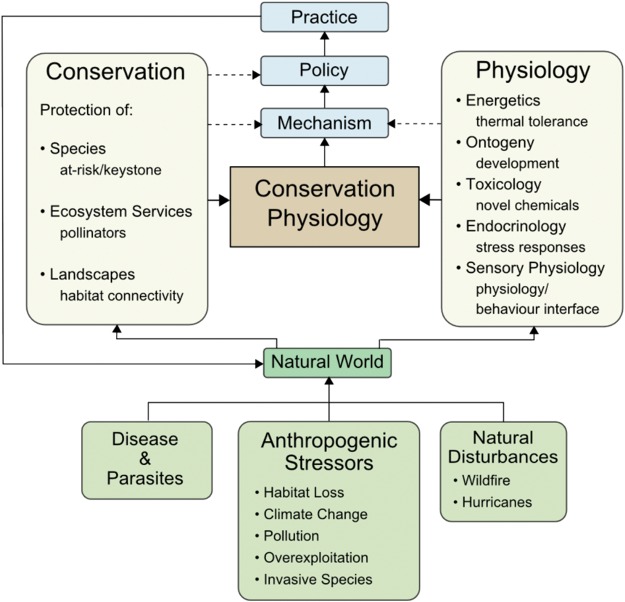
The interaction between conservation and physiology, with notable sub-concepts and examples of applications for both fields.

‘Conservation physiology’ integrates physiological perspectives into a broader conservation science (Fig. 1; Wikelski and Cooke, 2006). The merging of these two fields enables refinement of mechanistic knowledge that can be used to drive highly effective and specific policy recommendations. Conservation issues, broadly construed, include assessment of species and population viability, the anthropogenic threats that affect organisms, and intervention effectiveness. Prioritization of management interventions also falls under the umbrella of conservation. Physiology can be used to identify the sub-lethal and lethal effects that generate fitness decline. Thus, conservation physiology can be defined as a science that links global change effects on species abundance, dispersal and fitness to the physiological mechanisms that generate these declines and, in particular, the application of this knowledge to conservation efforts (Cooke *et al.*, 2013b).

Species are constrained in fitness and distribution by the range of environmental conditions they tolerate, which affects physiological performance and can alter population dynamics (Calow and Sibly, 1990; Spicer and Gaston, 1999; Ricklefs and Wikelski, 2002). Historically, human impacts on species and populations were most readily detected through assessment of declines, as with population censuses, which include measures of change in range size, distribution patterns, sex ratio and genetic diversity. These measures are time consuming and costly and, for some taxa, inaccurate, thereby detracting from conservation end-points (Hutchings and Baum, 2005). While the most immediately observed response to anthropogenic disturbance may be population declines, in most cases the response to any outside force starts at the level of a species' physiology. The extent to which monitoring-based approaches identify trends without clearly delineating their causes reduces the likelihood of achieving conservation management goals (Cooke and O'Connor, 2010). In many instances, conservation and management activities require frequent and rapid assessment of organismal response to interventions, yet decisions may be enacted in the absence of such scientific information (Salafsky *et al.*, 2002; Sutherland *et al.*, 2004; Dawson, 2011). Physiological measures can be incorporated into conservation as a means of overcoming these limitations (Wikelski and Cooke, 2006).

**Figure 2:**
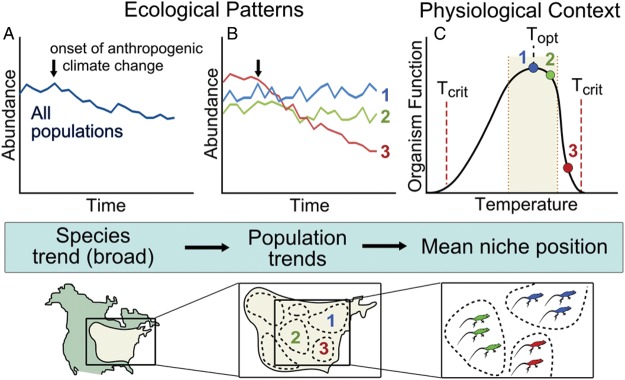
Differences in attribution of causal relationships between conservation (**A** and **B**) and conservation physiology studies (**B** and **C**). Physiological knowledge of a species (or other system of interest) can increase the precision with which mechanisms for responses are identified. Here, climate warming is causing a species of conservation concern to experience gradual decline, when taken as an average across all populations (A). However, an examination of distinct populations for this species (B) shows that population 3 is declining rapidly, while populations 1 and 2 are not. Knowledge of the thermal tolerance of this species can help to explain this pattern (C); individuals from population 1 are at the optimal temperature for the species and, therefore, the population has not experienced temperature-related declines. Individuals of population 2 are experiencing temperatures that are not optimal, and their function is not maximized; however, they are within the tolerable range of temperature for the species and, therefore, are not experiencing significant population decline. Population 2 is at risk of accelerating decline due to climate change in the near future. Individuals of population 3 are experiencing temperatures warmer than the optimal tolerable range for the species (shaded in beige), leading to deterioration of function at the individual level, which extrapolates to population-level decline. These individuals are experiencing sub-lethal effects and are approaching the critical temperature at which mortality occurs. Population 3 is at risk of local extinction, which could increase endangerment risk for the species. Active management of the population is warranted, and could involve translocation, removal of dispersal barriers, etc.

Physiological measurements provide additional mechanistic insights that may not be accessible from purely ecological studies (Fig. 2), enabling greater precision in detecting, attributing and predicting species' and individual responses to particular forms of environmental change (Wiens *et al.*, 1993; Helmuth *et al.*, 2005; Boyles *et al.*, 2011; Seebacher and Franklin, 2012). Many ecological principles are based, at least in abstract terms, on physiological processes but, in general, focus on broad-scale patterns that are generalizable across a range of environments and ecological contexts (Levin, 1992). Traditional techniques detect responses at the population level, or at the individual level between generations (i.e. when measuring reproductive output). Physiological response to environmental conditions is inter- and intra-specific (Spicer and Gaston, 1999; Cooke *et al.*, 2012), but may also be dependent on life stage (Pörtner and Farrell, 2008) or organismal responses that vary on diurnal or seasonal time scales (see Chown and Nicolson, 2004). Physiological techniques (i.e. monitoring stress hormones or whole-organism metrics of performance) can detect responses at the level of the individual at a very fine temporal resolution, as well as identify thresholds (Busch and Hayward, 2009) and vulnerability (Moritz and Agudo, 2013) to environmental stressors (and for the causal mechanism) that are relevant to the conservation issue. Given that optimization of physiological conditions relates to high fitness, while departures correspond to declines in organismal function and reproductive fitness (Arnold, 1983; Ricklefs and Wikelski, 2002; Pörtner and Farrell, 2008), physiology can be used to refine ecological mechanisms that have focused relevance to the conservation trajectories of species or populations.

The objective of this article is to outline a conceptual framework that details the role of conservation physiology in the larger conservation domain. This role includes discerning explicit physiological links between species fitness and environmental changes (Ricklefs and Wikelski, 2002; Tracy *et al.*, 2006), particularly where these have practical benefits for species' conservation outcomes through policy relevance (Cooke and Suski, 2008; Cooke and O'Connor, 2010; Cooke *et al.*, 2013b). Such contributions will then improve prospects for evidence-based decision-making (Sutherland *et al.*, 2004). The framework we propose here is intended to guide, but not limit, developments in this emerging discipline.

## Conservation physiology framework

**Figure 3:**
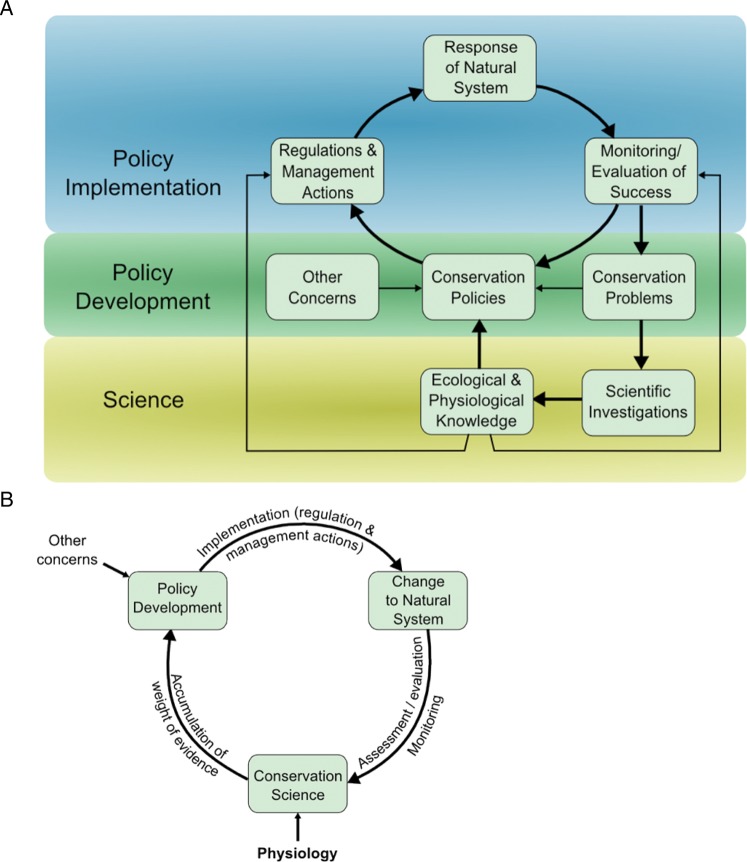
Process of interaction between conservation, physiology and policy. (**A**) Ways in which physiological knowledge can contribute to the conservation policy development and implementation process (adapted from Magnuszewski *et al.*, 2010). (**B**) Conservation, physiology and policy all provide feedback and input into each stage of the implementation and assessment process. Ongoing monitoring, assessment and evaluation increase the scientific weight of evidence and support decisive policy action.

Conservation physiology is an applied field that represents a solution-based approach to conservation and is a process of feedback between policy- and decision-makers and conservation physiologist practitioners. Physiology permits detection of incremental effects on species or population fitness, and this informs the decision-making process. This cycle of physiology informing conservation decision-making encourages an ongoing process of assessment, implementation, monitoring and evaluation. The integrated approach enables rapid modifications to conservation action based on changing conditions at any step in the process, because physiological knowledge identifies precise causal pathways for conservation issues and can detect sub-lethal effects (Fig. 3; adapted from Magnuszewski *et al.*, 2010).

Policy- and decision-makers should be considered the ultimate users of research findings in this context. As such, scientific findings need to translate into implementable solutions by being practical, repeatable and quantifiable. Cross-disciplinary collaboration and expertise improve integration and application of research findings within conservation management and policy (Meffe and Viederman, 1995). Policy-makers generate management decisions based on a combination of factors, including scientific research, societal views, normative values and socio-economic considerations (Gunningham *et al.*, 1998). These factors contribute to the identification of conservation problems and the determination of how problems are addressed through policy. Linking stressors with their concomitant effects on biodiversity (or population) status is an integral part of this process and enables policy-makers and conservation managers to incorporate explicit predictions of species response into management decisions.

Policy-makers may give a lower priority to scientific information when there are competing jurisdictional and socio-economic concerns (Findlay *et al.*, 2009). Improving the evidentiary weight of conservation research, as with the inclusion of physiology, can increase the likelihood that scientific information contributes to the policy process. When conservation issues have a clear physiological and mechanistic foundation, the scientific recommendations to policy-makers have higher levels of certainty, and non-evidence-based considerations that would lead to scientifically unsupported outcomes will less frequently play determining roles in policy development (Pullin *et al.*, 2004; Sutherland *et al.*, 2004). The conservation physiology framework is illustrated in Box A and Figure A. Our example of monarch butterfly (*Danaus plexippus*) decline across North America demonstrates the combined insights from ecological and physiological principles, which contribute to meaningful scientific recommendations that inform the conservation policy and decision-making process.

**Box A: Conservation physiology conceptual framework: monarch butterfly case study**

Justification for conservation physiology can be encapsulated elegantly by examining the plight of the monarch butterfly (*Danaus plexippus*; see Box Figure A). Ecological and conservation research demonstrates multiple aspects of life history as well as environmental requirements for the monarch butterfly. This species undergoes a multigenerational annual migration between southern Canada and Mexico. The monarch is designated as special concern in Canada (COSEWIC, 2013). Extensive loss of habitat in the overwintering and breeding grounds, climate change (Brower *et al.*, 2012) and increases in genetically modified crops, along with concomitant increase of pesticide use to control milkweed, its larval food source (Zalucki and Lammers, 2010; Pleasants and Oberhauser, 2013), have led to rapid and drastic population declines. Anthropogenic threats are distinct to each life stage of the monarch butterfly. Implementing solutions at each life stage often requires ecological, behavioural and physiological observations.

Physiological research has yielded additional, compelling insights that improve the conservation prospects for this species. An experimental, 2500 km westward displacement of butterflies at the commencement of their autumn migration determined that individuals have a high sensitivity to displacement, such as occurs with habitat fragmentation, climate change and loss of larval food sources. Due to the vector navigational system used, monarchs that are displaced from summering locations are unable to reorient towards Mexico. Refinement of migration direction occurs only at the culmination of the autumn migration through exogenous factors (Mouritsen *et al.*, 2013). Within the context of a conservation physiology framework, such information can be used to identify necessary conservation and policy action. Conservation research can identify whether populations are declining and can attribute these declines to specific anthropogenic threats. In this case, a purely conservation-based approach would generate a recommendation that habitat should be protected. Physiology identifies the precise physiological mechanism responsible for declines, leading to unambiguous solutions. In this specific instance, the vector navigation system used by monarch butterflies means that displacement from summering grounds translates into migration failure. Based on this research, conservation policies would focus on maintaining broad extents of habitat throughout the summering grounds. Given that monarchs undergo a multigenerational migration, there is a lag before effects of displacement (i.e. population loss) become apparent.

Conservation physiology builds on collaborative efforts from the fields of conservation, physiology and decision-makers using an iterative process of refinement that incorporates implementation, assessment and monitoring.

**Box Figure A:**
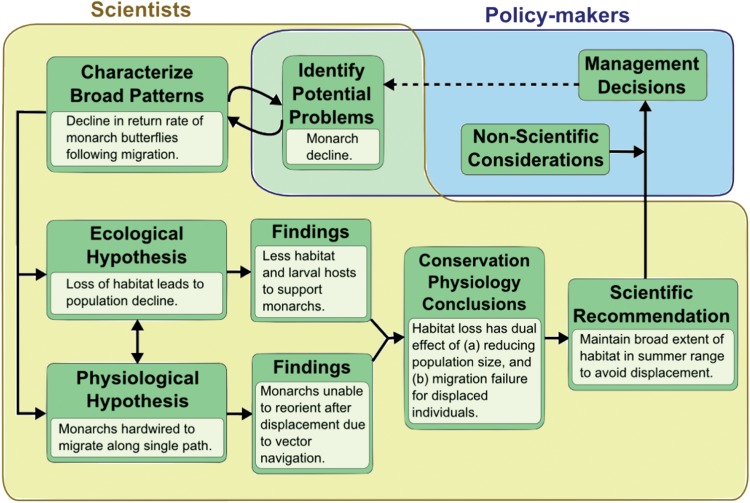
The general conservation physiology framework (dark green boxes) within the policy process (pale blue background), with supporting examples specific to monarch butterfly biology and management (light green boxes).

### Applying the framework

Generally, conservation physiology can be applied in any case where knowledge of an organism's physiology improves the ability to predict or manipulate ecological patterns and their conservation outcomes. These organisms include bacteria, plants and animals in both aquatic and terrestrial environments. Relevant applications of conservation physiology include informing suitability of management interventions (i.e. ecosystem restoration or species translocation), viability assessments for endangered population and species recovery, and threat assessments that predict the effects of current and future anthropogenic drivers of biodiversity decline and relevant interventions on distribution and abundance (Wikelski and Cooke, 2006; Cooke and Suski, 2008). For instance, all of the International Union for the Conservation of Nature–Conservation Measures Partnership (IUCN-CMP) threat categories can be examined using conservation physiology (see Table 1). Conservation physiology has broad utility in research as well, such as evaluation of competing ecological hypotheses by differentiating between expected physiological mechanisms (Tracy *et al.*, 2006). Additionally, physiological knowledge is now being used to assess the evolutionary basis for physiological adaptation in studies of phylogenetic niche conservatism and niche lability during climate change (Wiens and Graham, 2005). Among species with greater shared evolutionary history, trait-based responses to environmental changes are also likely to be shared, which may consequently lead to convergent responses to aspects of global change.

**Table 1:** Potential application of conservation physiology research to IUCN-CMP threats

IUCN-CMP threat category	Specific application	Physiological measures	Research
Residential and commercial development	Human land-use intensity effects on birds	Corticosterone and immunoglobulin	Chávez-Zichinelli *et al.* (2013)
Agriculture and aquaculture	Parasite incidence in aquaculture as an infective agent for wild salmon	Disturbance of ionoregulation	Brauner *et al.* (2012)
Energy production and mining	Aquatic pipeline crossing	Respiration, blood haematocrit and leucocrit, heart rate, etc.	Levesque and Dube (2007)
Transportation and service corridors	Effects of distance to road on bird species	Blood corticosterone levels	Dietz *et al.* (2013)
Biological resource use	Effects of logging and hunting on primates	Faecal glucorticoid metabolites	Rimbach *et al.* (2013)
Human intrusions and disturbance	Effects of tourism and food provisioning on endangered iguana	Dietary nutrition and endoparasitic infection	Knapp *et al.* (2013)
Natural system modifications	Mistiming of fire for red-backed fairy-wrens	Body mass and blood haemoglobin concentration	Murphy *et al.* (2010)
Invasive species, problematic species and diseases	Effects of season, humidity and sloughing on pathogens and infectious disease for frogs	Microbe abundance and recolonization rate	Cramp *et al.* (2014)
Pollution	Toxicity and mutagenicity post-oil spill	Photosynthetic activity of plankton, toxicity to microbes	Paul *et al.* (2013)
Geological events	Effects of volcanic mud exposure for fish	Phagocytic activity	Risjani *et al.* (2014)
Climate change and severe weather	Sensitivity to climate change across ontogenetic stages for endangered fish	Thermal and salinity limits, acclimatization states	Komoroske *et al.* (2014)

Abbreviation: IUCN-CMP, International Union for the Conservation of Nature–Conservation Measures Partnership.

In applying a conservation physiology framework, there are four main considerations. First, changes in a species' environment can be linked to species decline (as measured through performance, fitness or stress response, among others) using physiological measures. The physiological measure provides an explicit link between rates of change for physiological function and species decline. Second, these physiological mechanisms may differ by species, even within the same taxonomic group, although the phenotypic response, in terms of fitness, may be similar. Third, conservation physiology is an applied research discipline that can be used to tailor policy and management to the specific physiological response pathway. This occurs within the broader context of policy development and implementation. Fourth, the field benefits the wider conservation decision-making context by increasing the weight of available scientific evidence.

#### (i) Physiological links between species fitness and environmental changes

Knowledge of species' physiological responses has the potential to aid in devising effective conservation solutions. Wildlife corridor use is an example that illustrates the unique physiological response that a species may exhibit in response to environmental stressors. Corridors have a long history in conservation, yet may be ineffective because of inadequate baseline data on their utility for their target species (Chetkiewicz *et al.*, 2006). Research suggests that landscape use differs among individuals of a species based on physiological state. African elephants (*Loxodonta africana*) retreat to protected areas and corridors in response to human activities that cause physiological stress, as measured by faecal glucocorticoid metabolite hormones (Jachowski *et al.*, 2013). Where humans and elephants co-occur and protected areas are not available, human–elephant conflict is common. In some situations, this has led to detusking of elephants, which impacts social hierarchy and nutrition for these animals (Mutinda *et al.*, 2014). Strategic planning of corridors in regions with high human–elephant overlap is a more effective management tool and can provide elephants with a refuge that minimizes any potential conflict. Thus, physiological information can inform how and where protected areas are employed based on the level of anthropogenic pressure in the surrounding landscape.

#### (ii) Specificity of species–environmental links

Conservation efforts must often be tailored not only to the specific anthropogenic pressures but also to the species of concern and the physiological response mechanism. Thermal tolerance is a key determinant of species' fitness (Terblanche *et al.*, 2011) and distribution (Root, 1988; Sinclair *et al.*, 2003a), yet is governed by highly specific physiological mechanisms that vary by species. Climate change impacts are a rapidly developing area for conservation physiology studies (Monahan and Hijmans, 2008; Chown *et al.*, 2010), with the expectation that ranges for many species will expand poleward as temperatures warm at their cool thermal limits. For instance, freeze tolerance is a key strategy for ectotherms to survive sub-zero temperatures (Sinclair *et al.*, 2003a), such as for Isabella tiger moth (*Pyrrharctia isabella*) pupae. These organisms control the freezing process via a combination of ice-nucleating proteins and intra-cellular antifreeze (Marshall and Sinclair, 2012). For the Isabella tiger moth caterpillar, diminished snow cover due to climate warming increases exposure to prolonged sub-zero temperatures, yet because cold exposure induces freezing, metabolic expenditure is suppressed for a longer period, and emerging pupae have greater mass and higher fitness (Marshall and Sinclair, 2012). For freeze-tolerant ectotherms, there are two main causes of cold-induced mortality. Temperatures that drop below critical thresholds will cause severe tissue damage that translates into temperature-based northern range limits (Bale, 2002), but repeated cycles of freezing and thawing, as would be expected with increased weather fluctuations due to climate change, also cause tissue damage and lower survival (Marshall and Sinclair, 2011).

Other ectotherms, such as the invasive emerald ash borer (*Agrilus planipennis fairmaire*), are freeze avoidant (Crosthwaite *et al.*, 2011). These organisms use a combination of strategies, such as removal of ice-nucleating agents from cells and tissues, as well as increasing their supercooling capacity and using intra-cellular antifreeze to prevent ice crystal formation (Bale, 2002). Rapid lowering of temperature renders freeze-avoidant strategies ineffective (Sinclair *et al.*, 2003b). The emerald ash borer has undergone significant poleward range expansion since it was first observed in North America in 2002 (Venette and Abrahamson, 2010). The invasion front is limited by cold temperatures (<− 30°C), which reduce the intensity of ash infestation by decreasing emerald ash borer densities (DeSantis *et al.*, 2013).

In general and in the short-term, climate warming is likely to reduce barriers to poleward range expansion for both the Isabella tiger moth and the emerald ash borer by increasing overwintering survival and fitness (Crosthwaite *et al.*, 2011; Williams *et al.*, 2012). Long-term trends in warming will promote continued range expansion for the emerald ash borer, yet for the Isabella tiger moth this will eventually lead to reduced fitness if freezing cannot be maintained through the overwintering period. The physiological mechanisms that govern ecological responses for the Isabella tiger moth and emerald ash borer are markedly different. In practice, climatic extremes and rates of warming exert species-specific effects through distinctive physiological mechanisms (Bale and Hayward, 2010). Given that conservation focuses on altering outcomes for target organisms through either ameliorating conditions (and/or reducing barriers to fitness) for beneficial species or increasing barriers to fitness for invasive and pest species, knowledge of species-specific physiological mechanisms (and the manipulation thereof) has high applicability in policy.

#### (iii) Physiology as a method of promoting effective application of conservation

As an example of the utility of conservation physiology to policy, upstream relocation of Chinook salmon was once considered an effective method to enable fish bypass of water-diversion dams, and was incorporated into fish rescue strategies (see Mosser *et al.*, 2013). Lack of hydrological connectivity as well as increased water temperatures due to dam structures and climate warming contribute to high mortality for economically significant species. Conservation management decisions were previously based on the assumption that any intervention that improved connectivity would have a net benefit for the species (Hilborn, 2006). Fish relocation has low efficacy, but the reason was not determined until physiological impacts were examined. For salmon, cessation of migration occurs when upper thermal limits are exceeded, which may precipitate management interventions, such as upstream relocation. However, once upper thermal limits are exceeded, upstream relocation will have no impact, because the fish do not survive to reproduce (Mosser *et al.*, 2013). Among juvenile Chinook salmon, relocation hinders the physiological mechanisms responsible for homing and orientation during adult migration (Keefer *et al.*, 2008; Keefer and Caudill, 2012). Prior to these studies, capture and relocation was considered a viable conservation strategy for Chinook salmon (Mullen, 1987). In this case study, expensive management policies were implemented prior to the elaboration of mechanisms affecting relocation success rates. Conservation physiology research elucidated effects of relocation strategies, which led to entirely different management strategies, such as timing relocation efforts prior to temperatures exceeding critical limits, as well as decommissioning diversion dams and installing fish screens. The end result is a scientific recommendation that is far more likely to influence decision-makers, even though the costs of implementation are sometimes very high.

#### (iv) Informing decision-making through conservation physiology

Conservation physiology has the capacity to improve decision-making within the process of conservation policy development, implementation and assessment. Generally, conservation policies mandate particular management goals. The likelihood of conservation action (or inaction) reflects urgency, funding, jurisdiction and the potential impact of decisions or policies on stakeholders (Salafsky and Redford, 2013). Nevertheless, management interventions are unlikely to succeed if the causes of declines cannot be identified clearly. Conservation physiology contributes to potential management success by improving understanding of how stressors diminish the likelihood of species and individual survival (thereby identifying the proximate causes of population decline), predicting response to conservation actions and providing tools for evaluating and monitoring the effectiveness of a given action or regulation through time. Constraints to policy implementation (i.e. public views, economic considerations or competing interests, etc.) characterize the types of conservation physiology research that are considered feasible; however, researchers in the field should also strive to investigate what would be considered appropriate in the absence of constraints.

Physiological knowledge reduces uncertainty, which improves policy implementation. A minimal standard of evidence is required in any decision-making process where there is an assessment of risk. The acceptable standard of evidence changes based on perceived risk. Insufficient evidentiary strength and consistency is a common problem in conservation research and, inevitably, means that research fails to inform policy and management recommendations (Busch and Hayward, 2009). In cases where conservation strategies have had few marginal benefits (Ferraro and Pattanayak, 2006), this may be partly due to the lack of information about specific understanding of how and why species respond to human activities (Stewart *et al.*, 2005). Limited funding resources for conservation projects, when coupled with a low return on investment in terms of effectiveness (Sutherland *et al.*, 2004), leave room for the decision process to be driven by values and economic considerations that argue against action (Findlay *et al.*, 2009; Mooers *et al.*, 2010).

Weight of evidence represents a systematic approach to quantifying uncertainty (Sutherland *et al.*, 2004). To generate recommendations that advance conservation objectives, research findings must first contribute to a minimal weight of evidence (Thompson *et al.*, 2005) and, second, contribute to transparent evaluation of implemented recommendations (Ferraro and Pattanayak, 2006; Guyatt *et al.*, 2008). Effective study design is one of the most critical factors used to generate the high-impact evidentiary standards and evaluation of outcomes (Sutherland *et al.*, 2004; Carey, 2005). Enhanced evidentiary quality occurs with consideration of effect size, consistency of results across multiple studies, precision and publication bias (for a more detailed discussion of these and other considerations, see Guyatt *et al.*, 2008). Conservation physiology has the potential to increase the scientific contribution to policy development by providing an experimental or pseudo-experimental design that identifies not only the mechanism for effects but also the precise relationship between the rate of environmental change and species fitness (Carey, 2005; Cooke *et al.*, 2013a). In doing so, conservation physiology promotes research application in a management and policy context.

## Challenges

Conservation physiology, as a new field, faces a number of hurdles; among the most consequential is the need to improve the applicability of physiological data and measurements to conservation. An additional challenge, where theoretical insights may be particularly critical, is the need to discover ways to ‘scale up’ from physiological observations to ecological pattern (Levin, 1992; Cooke and O'Connor, 2010; Cooke *et al.*, 2014). Differences in the scale of investigation between the two fields can lead to difficulties of extrapolation, particularly if the examined end-point varies substantially within and between populations and species. Finally, translating discovery at the conservation and physiological interface into management application is the final and, arguably, best test of success for this field. Like conservation biology itself, conservation physiology is a mission-oriented discipline (e.g. Soulé, 1985).

Physiological measurements and tools necessary to overcome such hurdles should be non-invasive, non-lethal and, ideally, involve rapid assessment (Cooke and O'Connor, 2010). Obtaining reliable baseline data is problematic in many fields; however, two options are to improve data accessibility through data sharing (Wolkovich *et al.*, 2012) and to employ time series and rate-of-change study designs (Cooke and O'Connor, 2010). Furthermore, the scope of effectiveness for conservation physiology improves if individual- and population-level effects (as measured through physiological investigations) are linked to the species and communities of conservation concern.

Improved education for physiologists, conservationists and policy-makers on the policy process and conservation needs is essential and will foster higher impact collaboration (Cooke and O'Connor, 2010). The inclusion of managers and policy-makers in conservation physiology research will improve stakeholder and individual participation and the likelihood that research results will be applied. While there need not be an expectation that every conservation physiological research outcome will find direct policy application, policy relevance and impact should, nevertheless, remain a key consideration in conservation physiology research. To facilitate this, research should be accessible to conservation practitioners (Pullin *et al.*, 2004; Stewart *et al.*, 2005) and a greater emphasis placed on interpretive scientific skills.

Collaboration is an integral component of conservation physiology but is not without attendant challenges. Overlap in terminology changes for physiology and conservation did not generally increase following the initial coining of the term ‘conservation physiology’ (Lennox and Cooke, 2014). When both sides of the conservation physiology discipline can view the findings of the other, it leads to a mutual awareness of contributions (Sutherland *et al.*, 2013), a need highlighted and partly addressed by the newly created journal, *Conservation Physiology*. The highly specialized knowledge base required for physiology, conservation, and policy and management decision-making demands collaborative efforts. This, in turn, generates strong and relevant scientific knowledge that can inform conservation decision-making.

## Conclusions

Here, we have outlined a conceptual framework for merging conservation and physiology that we argue will yield improved conservation decision-making. Within the broader suite of processes that make up conservation policy development and implementation, this application of physiological knowledge is most useful to informing development of the following aspects: (i) overall policies that respond to a conservation problem; (ii) on-the-ground adaptive management actions that effectively accomplish the conservation objectives mandated by those policies; and (iii) evaluation tools and techniques that characterize the effectiveness of both of these at mitigating conservation problems. The strength of the conservation physiology framework arises from an integrative approach with an applied focus. This translates into improved dialogue and input between practitioners of conservation, physiology and policy, where each informs the design, conduct and implementation of conservation physiology research.

Given that physiological research investigates causal response mechanisms to changes in optimal environmental conditions, where shifts in organismal condition relative to physiological requirements affect overall functioning and fitness (Tracy *et al.*, 2006), conservation physiology can rigorously inform the decision process for policy and management (Carey, 2005). Conservation and policy needs identify critical research questions for conservation physiologists; physiology reveals the mechanistic underpinnings of behaviour and performance (Cooke *et al.*, 2014), thereby identifying new policy needs to promote conservation. In a world of pervasive human influence on the natural world, there is a growing need for conservation research to produce strong and decisive evidence for the consequences to natural systems (Rudd *et al.*, 2011). Conservation physiology is uniquely poised to meet this challenge.

## Funding

This work was supported by the Natural Sciences and Engineering Research Council (Discovery Grant Program, S.J.C. and J.T.K.; Post Doctoral Fellows Program, C.M.O.; Canada Graduate Scholarship, C.M.R.) and by the Ontario Graduate Scholarship Program (L.E.C.). This work was further supported by the Canada Research Chair Program (S.J.C.), the University Research Chair Program at the University of Ottawa (J.T.K.) and the University of Ottawa Excellence Scholarship Program (L.E.C. and C.M.R.). Additional funding sources include the E.B. Eastburn program (C.M.O.) and Conservation International (D.L.).
